# Thioredoxin reductase: A novel, independent prognostic marker in patients with hepatocellular carcinoma

**DOI:** 10.18632/oncotarget.3785

**Published:** 2015-04-23

**Authors:** Chunyan Li, Yan Peng, Binglang Mao, Kun Qian

**Affiliations:** ^1^ Department of Endocrine and Breast Surgery, The First Affiliated Hospital of Chongqing Medical University, Chongqing 400016, China; ^2^ Biotherapy Center, The First Affiliated Hospital of Chongqing Medical University, Chongqing 400016, China; ^3^ Department of Critical Care Medicine, The First Affiliated Hospital of Chongqing Medical University, Chongqing 400016, China; ^4^ The Medical Examination Center, The First Affiliated Hospital of Chongqing Medical University, Chongqing 400016, China; ^5^ Department of Gastrointestinal Surgery, The First Affiliated Hospital of Chongqing Medical University, Chongqing 400016, China

**Keywords:** hepatocellular carcinoma, thioredoxin reductase, prognosis, recurrence, chinese

## Abstract

Here we found that hepatocellular carcinoma (HCC) patients with recurrence outcome and nonsurvivors had significantly increased thioredoxin reductase (TrxR) serum levels on reoperation (*P* < 0.0001 and *P* < 0.0001). Multivariate regression analysis adjusted for common risk factors showed that TrxR was an independent predictor of recurrence (hazard ratios [HR] = 4.19; 95% confidence intervals [CI]: 3.21–7.08) and overall survival (HR = 5.56; 95% CI: 3.42–10.21). The area under the receiver operating characteristic curve of TrxR was 0.837 (95% CI, 0.794–0.881) for recurrence outcome and 0.901 (95% CI, 0.869–0.933) for mortality, which was superior to high-sensitivity-C-reactive protein and a-fetoprotein (*P* < 0.001). The preoperative serum TrxR level is an independent and significant indicator predictive of poor prognosis and early recurrence in patients with HCC, which offering reliable information for predicting survival.

## INTRODUCTION

Hepatocellular carcinoma (HCC) is one of the most frequent malignant tumors and is the second most common cause of cancer death in China [1]. Chinese HCC patients account for > 55% of new HCC cases worldwide. A 10-year survey (1990–2001) indicated that HCC ranks first among chronic diseases for the social cost and burden in the WHO disability-adjusted life year list in China [2]. The 5-year survival rate of all HCC is < 5%, placing it among the cancers with the worst prognosis [3].

Thanks to remarkable advances in surgical techniques and perioperative patients' management, hepatic resection has been established as one of the most effective treatments. It also should be noted that HCC is a complex and heterogeneous disease involving several etiological agents that exert differential effects on the molecular pathways involved. However, surgery, including transplantation, remains the only curative modality for HCC accompanied with high frequent recurrences and low 5-year survival rate ranging from 17% to 53% [4]. Reliable markers to identify patients who are at high-risk for early death and recurrence would be beneficial. Therefore, it is very important to detect this disease and the recurrence at its earlier period.

Several tumor markers for HCC, such as alpha fetoprotein (AFP) [5], retinol-binding protein 4 [6], desgamma-carboxy prothrombin time (DCP) and AFP-leptin 3 (AFP-L3) [7], have been identified as potential candidates to evaluate the prognosis of patients with HCC. However, they are not always sufficient for prediction of recurrence and prognosis. In this regard, the search for innovative biomarkers capable of identifying high-risk patients and of modulating cancer treatment options is still actively ongoing.

Redox dysregulation originating from metabolic alterations and dependence on mitogenic and survival signaling through reactive oxygen species represents a specific vulnerability of malignant cells that can be selectively targeted by redox chemotherapeutics [8]. Redox control has emerged as a fundamental biological control mechanism. One of the major redox control systems is the thioredoxin system comprised of thioredoxin (TRX) and thioredoxin reductase (TrxR) [9]. Like all members of the enzyme family, TrxR contains a redox active disulfide adjacent to the flavin ring [10]. The mammalian TrxR are a family of selenium-containing pyridine nucleotide-disulphide oxidoreductases with mechanistic and sequence identity, including a conserved-Cys-Val-Asn-Val-Gly-Cys-redox catalytic site, to glutathione reductases [11]. TrxRs catalyse the NADPH-dependent reduction of the redox protein TRX, as well as of other endogenous and exogenous compounds. There are currently two confirmed forms of mammalian TrxRs: TrxR1 and TrxR2.

Secretion of TrxR under conditions of oxidative stress and inflammation has been observed from many normal and neoplastic cells [12]. Berggren et al. [13] found that TrxR protein and activity were increased an average of 2-fold in human colorectal tumors compared to normal mucosa. The level of TrxR in tumor cells is often 10-fold or even greater than in normal tissues, and tumor proliferation seems to be crucially dependent on an active thioredoxin system [14]. TrxR over-expression has been reported in several malignancies (breast, prostate, colorectal and hepatocellular carcinomas) and may be associated with aggressive tumor growth and poor survival [9, 13, 15]. In sharp contrast, the possible presence of TrxR in serum has remained much less elucidated. Herein, the aims of this study were to determine if TrxR was detected in the serum, and to establish the long-term prognostic value of early measurement of serum TrxR levels in Chinese patients with HCC after curative resection.

## RESULTS

### Patient characteristics

From 426 screened HCC patients underwent curative hepatic resection, 344 patients (22 with malignancy other than HCC, 10 patients who died within 30 days after surgery [operative mortality], 19 without informed consent, 9 with systemic infections and 22 lost follow-up were not analyzed) were included and completed follow-up. The end of follow-up was either the time of last follow-up (36 months after operation) or death. The baseline characteristics of the 344 patients presenting with HCC were described in Table 1.

**Table 1:** The baseline characteristics of the patients with HCC and control

	HCC	Control^a^	*P*
N	344	200	
Age (IQR, years)	58 (47–66)	58 (48–65)	0.783
Males (%)	59.9	60.0	0.912
Aetiology (N)^a^			0.321
HBV	284	161	
HCV	57	36	
Combination (HBV+HCV)	10	6	
Alcohol	9	6	
Other	4	3	
Tumor size (IQR, cm)	6.9 (3.8–10.8)	—	
Presence of PVT (%)	118 (34.3)	—	
Venous invasion (%)	73 (21.2)	—	
Child-Pugh (%)			
Class A	212 (61.6%)	—	
Class B	95 (27.6%)	—	
Class C	37 (8.5%)	—	
Tumour stage^b^			
I	104 (30.2%)	—	
II	109 (31.7%)	—	
III	89 (25.9%)	—	
IV	42 (12.2%)	—	
No. of nodules (%)			
1	177 (51.5)	—	
> 1	167 (48.5)	—	
Nodule size (%)			
≤ 3 cm	76 (22.1)	—	
> 3 cm	268 (77.9)	—	
Laboratory findings (IQR)			
Platelet counts (×10^3^/mm^3^)	155 (126–198)	163 (131–203)	0.112
ALT (IU/L)	107 (47–198)	132 (59–226)	0.012
AST(IU/L)	118 (43–204)	137 (62–244)	0.018
Total bilirubin (mg/dL)	1.01 (0.88–1.44)	1.17 (0.96–1.87)	0.007
Albumin (g/dL)	36.3 (33.2–40.8)	36.7 (33.5–41.1)	0.218
Prothrombin time (%)	14.2 (11.2–15.6)	13.8 (12.1–14.9)	0.033
Hs-CRP (ng/ml)	0.48 (0.21–1.55)	0.35 (0.18–0.76)	0.003
αFP (ng/ml)	80.5 (29.5–156)	15.8 (7.4–30.7)	<0.001
TrxR (U/ml)	16.1 (9.0–23.2)	5.2 (3.3–7.2)	<0.001

a 200 control cases were included. 100 patients with liver cirrhosis, 100 patients with chronic liver disease

b Tumour was staged accordingly to the American Liver Tumour Study Group modified TNM staging classification

IQR = interquartile ranges; HBV = hepatitis B virus; HCV = hepatitis C virus; ALT = alanine aminotransferase; AST = Aspartate transaminase; PVT = portal vein thrombosis; AFP = α-fetoprotein; Hs-CRP = High-sensitivity-C-reactive protein; TrxR = Thioredoxin reductase.

Overall median age was 58 (IQR, 47–66) years and 59.9% were men. At diagnosis, 212 (61.6%) patients were classified as Child-Pugh class A, 95 (27.6%) as class B and 37 (8.5%) as class C. There were 104 (30.2%) patients at TNM stage I, 109 (31.7%) at stage II, 89 (25.9%) at stage III, and 42 (12.2%) at stage IV. The median tumor size was 6.9 (3.8–10.8) cm and the number of patients with solitary tumor was 177 (51.5%). Each case of presence of PVT and venous invasion was 118 (34.3%) and 73 (21.2%), respectively. The median levels of serum AFP and Hs-CRP were 80.5 (IQR, 29.5–156) ng/ml and 0.48 (0.21–1.55) mg/dL, respectively. Recurrence was found in 105 patients (30.5%) and 111 patients died, thus the mortality rate was 32.3%. The causes of death were as follows: HCC recurrence (*n* = 98; 88.3%), liver failure (*n* = 7; 6.3%), and other causes (*n* = 6; 5.4%).

### TrxR and clinical variables

**Figure 1:**
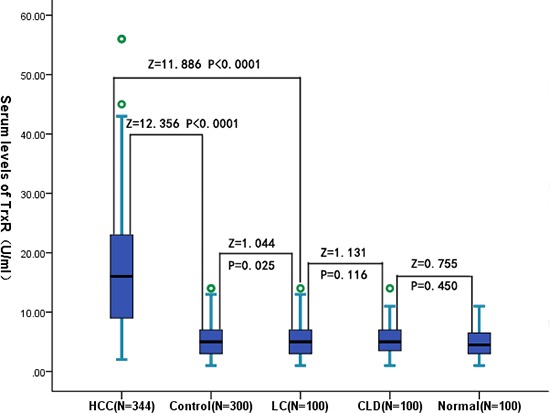
Box plot for serum TrxR values in the studied groups The box indicates the 25th and 75th percentile of the data, and the middle line, the median. A line extends from the minimum to the maximum value, excluding outliers that are displayed as separate points. CLD = chronic liver diseases; LC = liver cirrhosis; HCC = hepatocellular carcinoma.

**Figure 2:**
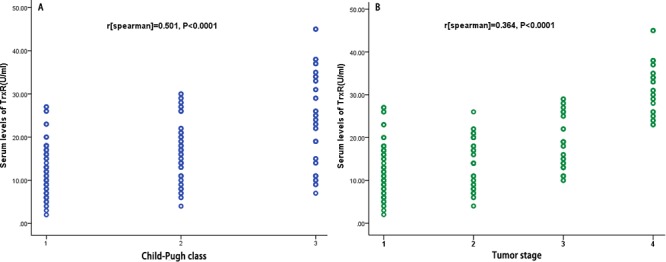
Correlation between the TrxR serum levels and other factors **A.** Correlation between the TrxR serum levels and Child-Pugh class (1 = A; 2 = B; 3 = C); **B.** Correlation between the TrxR serum levels and Tumor stage.

**Figure 3:**
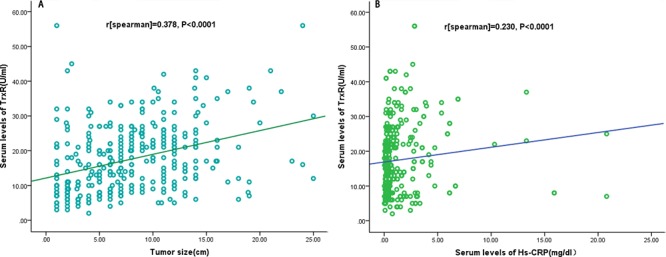
Correlation between the TrxR serum levels and other factors **A.** Correlation between the TrxR serum levels and tumor size; **B.** Correlation between the TrxR serum levels and Hs-CRP.

The results indicated that the serum TrxR levels were significantly (*P* < 0.0001) higher in HCC patients as compared to controls [16.1 (IQR, 9.0–23.2 U/ml) *vs*. 5.1 (IQR, 3.0–7.1 U/ml); Figure 1]. Serum TrxR levels were also significantly higher in HCC when compare with LC and CLD (Figure 1). There was no significantly difference among the three groups of control cases (Figure 1). In addition, the relationship of TrxR with Child-Pugh class and tumor characteristics (TNM), which were the two major determinants of the prognosis of patients with HCC, were evaluated. There was a significant correlation between TrxR and Child-Pugh class or tumor stage (*r* [spearman] = 0.501, *P* < 0.0001; *r* = 0.364, *P* < 0.0001, respectively). As shown in Figures 2a and 2b, the level of TrxR tended to increase as liver disease progressed from Child-Pugh class A to C as well as tumor stage from I to IV. There was still a significant positive correction between TrxR serum levels and Child-Pugh class (*P* = 0.006) or tumor stage (*P* = 0.002), using ordered logistic regression after multivariate adjustment for possible confounders. There was a significantly correlation between TrxR levels and tumor size (*r* = 0.378, *P* < 0.0001; Figure 3a), and the level of TrxR elevated as the tumor size increased. In addition, there was a weak but significant positive correlation between TrxR and Hs-CRP (*r* = 0.230, *P* < 0.0001; Figure 3b). We also found that there was a positive correlation between TrxR and venous invasion (*r* = 244, *P* < 0.0001). Statistical analysis here revealed no influence of age, sex, infection time, etiology, family history, HbsAg, HBV or HCV copies, ALT, AST, TB, PT and AFP on TrxR in HCC patients (*P* > 0.05, respectively).

### TrxR and 36-month recurrence

**Figure 4:**
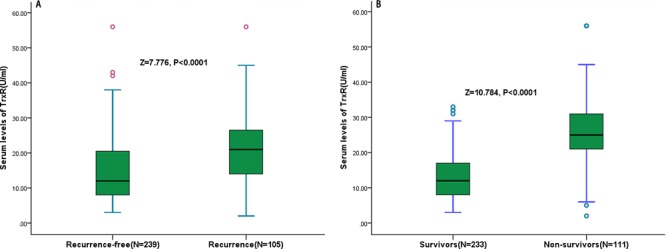
Serum TrxR levels in different groups **A.** Serum levels of TrxR in patients with recurrence and recurrence-free outcomes; **B.** Serum levels of TrxR in survivors and non-survivors.

In the 105 patients with recurrence, serum TrxR levels were higher compared with those in patients with a recurrence-free [22.5 (IQR, 11.4–26.9) U/ml *vs*. 11.8 (IQR, 7.5–20.1) U/ml; *p* < 0.0001; Figure 4a). The relation of TrxR and other factors with recurrence was investigated with the use of Cox regression models. In regression analysis, we calculated the HR of log-transformed TrxR levels as compared with AFP, Hs-CRP and other factors as presented in Table 2. With an unadjusted HR of 10.23 (95% CI, 4.14–30.76; *P* < 0.0001), TrxR had a strong association with recurrence. After adjusting for all other significant outcome predictors, TrxR remained an independent recurrence predictor with an adjusted HR of 4.19 (95% CI, 3.21–7.08; *P* < 0.0001). In addition, tumor size, TNM stage, Child-Pugh class and laboratory findings, such as AFP and Hs-CRP remained significant recurrence predictors (Table 2).

**Table 2:** Univariate and multivariate analysis for recurrence and OS by Cox regression models

Parameter	Univariate Analysis			Multivariate Analysis		
	HR	95% CI	*P*	HR	95% CI	*P*
Predictor: recurrence						
Age(>60 years)	1.17	1.08–2.11	0.017	1.13	1.04–1.96	0.027
Sex (male)	0.83	0.50–2.11	0.713	—		
Cause(HBV)	1.64	0.95–2.87	0.076	—		
Viral status	2.30	1.20–4.21	0.011	2.01	1.09–4.09	0.029
Tumor number	1.29	1.02–2.31	0.673	—		
Tumor size	1.78	1.31–3.24	0.021	1.54	1.24–2.91	0.038
Venous invasion	2.11	1.49–4.99	<0.001	1.87	1.32–3.06	0.002
TNM stage	3.23	1.46–7.32	0.001	2.08	1.22–4.15	0.006
Child-Pugh class	2.21	1.66–4.87	<0.001	2.01	1.51–4.31	<0.001
Presence of PVT	3.55	2.62–4.87	<0.001	1.78	1.21–2.65	0.003
Presence of extrahepatic	4.04	3.01–5.77	<0.001	1.85	1.28–2.62	0.001
AFP(>20 ng/ml)	2.07	1.42–3.01	0.008	1.88	1.27–3.98	0.011
Hs-CRP(>0.4 mg/dl)	2.18	1.71–4.45	<0.001	1.79	1.36–3.39	0.008
TrxR^a^	10.23	4.14–30.76	<0.001	4.19	3.21–7.08	<0.001
Predictor: OS						
Age(>60 years)	1.69	1.34–3.04	0.019	1.44	1.13–2.56	0.036
Sex (male)	0.69	0.35–1.34	0.241	—		
Cause(HBV)	1.54	1.06–3.21	0.097	—		
Viral status	1.24	1.07–3.32	0.332	—		
Tumor number	1.42	1.02–1.98	0.110	—		
Tumor size	1.99	1.25–4.09	0.012	1.79	1.18–3.04	0.018
Venous invasion	2.11	1.49–3.59	<0.001	1.57	1.21–2.87	0.002
TNM stage	2.24	1.22–4.02	<0.001	2.11	1.42–3.29	0.001
Child-Pugh class	2.06	1.42–2.91	<0.001	2.00	1.55–4.26	<0.001
Presence of PVT	3.95	2.90–5.23	<0.001	1.66	1.08–2.75	0.022
Presence of extrahepatic	3.05	2.22–4.19	<0.001	1.62	1.13–2.26	0.008
AFP(>20 ng/ml)	2.19	1.09–4.32	0.028	1.94	1.10–3.77	0.038
Hs-CRP(>0.4 mg/dl)	3.06	1.66–5.63	<0.001	2.49	1.54–4.06	<0.001
TrxR^a^	16.15	6.11–39.65	<0.001	5.56	3.42–10.21	<0.001

a increase per log unit(thus, a log-transformed increase of 1 corresponds to a TrxR increase of 10 U/mL)

OS = Overall survival; CI = confidence intervals; HR = hazard ratios; HBV = hepatitis B virus; PVT = portal vein thrombosis; AFP = α-fetoprotein; Hs-CRP = High-sensitivity-C-reactive protein; TrxR = Thioredoxin reductase.

With an AUC of 0.837 (95% CI, 0.794–0.881), TrxR showed a significantly greater discriminatory ability as compared with Hs-CRP (AUC, 0.649; 95% CI, 0.586–0.712; *P* < 0.001) and AFP (AUC, 0.584; 95% CI, 0.519–0.659; *P* < 0.001), while was in the range of Child-Pugh class (AUC, 0.827; 95% CI, 0.799–0.878; *P* = 0.658). Interestingly, the combined model (TrxR/AFP/Hs-CRP) improved those factors alone (AUC of the combined model, 0.883; 95% CI, 0.819–0.947). This improvement was stable in an internal 5-fold cross validation that resulted in an average AUC (standard error) of 0.837 (0.022) for the TrxR and 0.883 (0.016) for the combined model, corresponding to a difference of 0.046 (0.006). In addition, a model containing known risk factors plus TrxR compared with a model containing known risk factors without TrxR showed a greater discriminatory ability (Table 3).

**Table 3:** Prediction of recurrence recurrence and OS

Parameter	AUC	95% CI		*p*
Prediction of recurrence				
TrxR	0.837	0.794	0.881	
Child-Pugh class	0.827	0.799	0.878	0.658
Tumour stage	0.785	0.702	0.813	0.007
Age	0.562	0.504	0.632	<0.001
AFP	0.584	0.519	0.659	<0.001
Hs-CRP	0.649	0.586	0.718	<0.001
Combined score^a^	0.883	0.819	0.947	0.003
Combined score^b^	0.865	0.732	0.913	0.008
Combined score^c^	0.924	0.827	0.961	<0.001
Prediction of OS				
TrxR	0.901	0.869	0.933	
Child-Pugh class	0.802	0.717	0.869	0.002
Tumour stage	0.799	0.714	0.856	0.001
Age	0.551	0.503	0.624	<0.001
AFP	0.586	0.525	0.647	<0.001
Hs-CRP	0.679	0.618	0.740	<0.001
Combined score^a^	0.933	0.879	0.977	<0.001
Combined score^b^	0.874	0.797	0.904	<0.001
Combined score^c^	0.947	0.879	0.981	<0.001

AUC = area under the curve; CI = confidence interval; Hs-CRP = High-sensitivity-C-reactive protein; AFP = α-fetoprotein; TrxR = Thioredoxin reductase

a including TrxR/Hs-CRP/AFP.

b including Child-Pugh class/Tumour stage/age/AFP/Hs-CRP.

c including Child-Pugh class/Tumour stage/age/AFP/Hs-CRP/TrxR.

### TrxR and 36-month mortality

At 3 years, 111 HCC patients (32.3%) had died. Non-survivors had significantly higher TrxR levels than survivors (25.6 [IQR, 21.0–31.5] U/ml *vs.* 11.3 [IQR, 7.2–17.9] U/ml; *P* < 0.0001; Figure 4b). Similarly, the relation of TrxR and other factors with OS was investigated with the use of Cox regression models. In logistic regression analysis, we calculated the HR of log-transformed TrxR levels as compared with AFP, Hs-CRP and other factors as presented in Table 2. With an unadjusted HR of 16.15 (95% CI, 6.11–49.65; *P* < 0.0001), TrxR had a strong association with OS. After adjusting for all other significant predictors, TrxR remained an independent OS predictor with an adjusted HR of 5.56 (95% CI, 3.41–10.21; *P* < 0.0001). Again, tumor size, TNM stage, Child-Pugh class and laboratory findings, such as AFP and Hs-CRP remained significant OS predictors (Table 2).

Similarly, with an AUC of 0.901 (95% CI, 0.869–0.933), TrxR showed a significantly greater discriminatory ability as compared with Child-Pugh class (AUC, 0.802; 95% CI, 0.717–0.869; *P* < 0.001), Hs-CRP (AUC, 0.679; 95% CI, 0.618–0.740; *P* < 0.001) and AFP (AUC, 0.586; 95% CI, 0.525–0.647; *P* < 0.001). Interestingly, the combined model (TrxR/AFP/Hs-CRP) also improved those factors alone (AUC of the combined model, 0.933; 95% CI, 0.879–0.977). Again, this improvement was stable in an internal 5-fold cross validation that resulted in an average AUC (standard error) of 0.901 (0.016) for the TrxR and 0.933 (0.010) for the combined model, corresponding to a difference of 0.032 (0.006). In addition, a model containing known risk factors plus TrxR compared with a model containing known risk factors without TrxR showed a greater discriminatory ability. Table 3.

**Figure 5:**
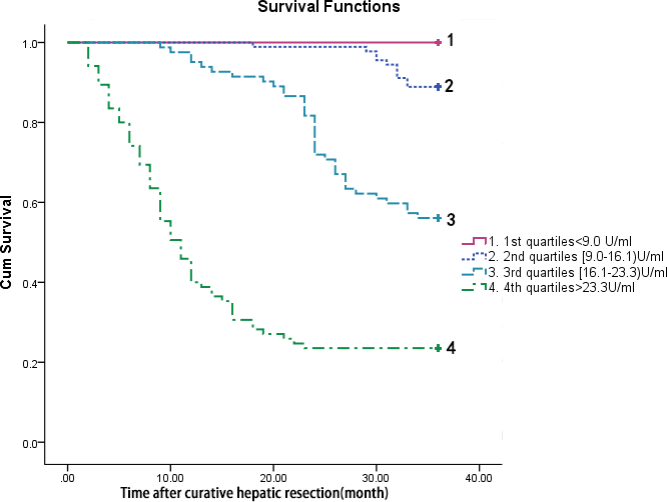
Kaplan–Meier survival based on TrxR quartiles Time to death was analysed by Kaplan–Meier curves based on TrxR quartiles. Patients in the lower two quartile (TrxR < 9.0 U/ml and TrxR between 9.0 and 16.1 U/ml) had a minor risk of death compared to patients with TrxR levels in the higher two quartile (TrxR > 23.3 U/ml and TrxR between 16.1 and 23.3 U/ml, *P* < 0.0001).

The time to death was analyzed by Kaplan–Meier survival curves based on serum TrxR quartiles. Patients in the upper two quartiles had a higher risk of death compared to patients with TrxR levels in the lower two quartile (Figure 5).

## DISCUSSION

Although surgical resection and liver transplantation provided valid approaches to treat HCC, the 5-year recurrence rate after curative resection is still high [6]. In light of the poor prognosis in advanced HCC, rapidly measurable biomarkers to predict illness development, recurrence and mortality are pivotal for optimized care and allocation of healthcare resources. Therefore, we designed the current study to evaluate the significance of preoperative TrxR serum levels on recurrence and survival in patients with HCC after hepatic resection.

To the best of our knowledge, in this study, we firstly demonstrated that the preoperative serum TrxR level may be an independent and significant prognostic indicator in patients with HCC after curative hepatic resection. Among all of the factors investigated in this study, the TrxR level proved to be the most powerful indicator, and the combined model (TrxR/AFP/Hs-CRP) improved those factors alone. In addition, the level of TrxR tended to increase as liver disease progressed from Child-Pugh class A to C as well as tumor stage from I to IV. Moreover, TrxR was found to strongly correlate with a number of well-known tumor-related prognostic and recurrent factors. Importantly, TrxR still was a prognostic marker even after correcting for above confounding factors.

Previous literatures on thioredoxin system (TRX/TrxR) in HCC had been reported. Miyazaki et al. [16] suggested that measurement of serum of TRX might be a useful clinical parameter when HCC was suspected, while Choi et al. [17] found that alterations in cellular redox status by enhanced expression of TrxR2 might be associated with the formation and development of HCC. In addition, the role of TrxR in the HCC progression had been studied. One study shows that TrxR1 protects against chemically induced hepatocarcinogenesis via the control of the cellular redox state, whereas its role in promoting this type of cancer is minimal [18]. Duan et al. [19] discovered that gambogic acid [GA] may bind with TrxR1 to elicit oxidative stress and eventually induce apoptosis in human HCC SMMC-7721 cells. Importantly, over-expression of functional TrxR1 in cells attenuates the cytotoxicity of GA, whereas knockdown of TrxR1 sensitizes cells to GA. Similarly, Gan et al. [20] reported that inhibition of TrxR over-expression can suppress the growth of human HCC SMMC-7721 cells.

The most important adverse prognostic factor for liver resection and transplantation in HCC has been found to be microscopic venous invasion [21]. However, microscopic portal invasion is not diagnosed preoperatively, and is revealed only by pathological examination. In fact, these are well known indicators for poor prognosis and early tumor recurrence in patients with HCC, for instance, glypican 3 [22], MicroRNA-29 [23], hyaluronic acid [24], Ski-interacting protein [25], interleukin-6 [26], VEGFR2 [27], IL-17 and IL-17RE [28], G2890 N-glycan [29], GGT [30] and miR-24-3p [31]. Zhu et al. [7] indicated that serum YKL-40 was an independent prognostic factor for overall and RFS in HCC patients receiving curative resection. It has been reported that AFP is an independent predictor of prognosis for HCC, even in patients who have received a hepatectomy [32]. However, it is well known that AFP may increase in some patients with acute and chronic hepatitis without HCC [33], and that the elevation of AFP correlates with inflammation of background disease and hepatocyte regeneration [34]. Therefore, the AFP levels do not influence patient survival and recurrence. In another study, Oh et al. [35] suggested that CRP and neutrophil-to-lymphocyte ratio are important prognostic biomarkers for HCC. Similarly, Kinoshita et al. [36] demonstrated that the pretreatment conventionally measured CRP was associated with tumor progression and reduced liver function and can be considered as an independent marker of poor prognosis in patients with HCC. In our study, we found that elevated serum TrxR was a novel, independent prognostic factor for OS and recurrence in Chinese HCC patients receiving curative resection, and may serve as a future biomarker. Importantly, the prognostic value of TrxR was better than Hs-CRP and AFP.

In our study, we found that the serum TrxR levels were significantly (*P* < 0.0001) higher in HCC patients as compared to controls. Whether higher circulating TRX level is an accelerator or only be a marker of HCC remains uncertain. Sun et al. [37] found that serum TrxR levels were robustly elevated in mouse models of chemically induced liver injury. Thus, the TrxR in the serum may come from ruptured cancer. Mollbrink et al. [15] showed that TrxRs increased in HCCs as compared to the respective surrounding liver, which supported our above hypothesis. However, in our study, TrxR measurements were performed after the HCC and may not accurately reflect pre-HCC exposure. Therefore, we could not confirm the high level of TrxR found in serum be due to that coming from ruptured cancer or endothelial cells or from extra cellular proteins. Further study should be considered.

The mechanism by which a systemic redox dysregulation response reflected by an elevation of TrxR serum levels might influence recurrence or survival in patients with cancer is not clear. Some studies have compiled the possible roles of CRP in cancer development. The significant prognostic role of TrxR is supported by the evidence that the levels of marker displayed a linear relationship with the progressing stage of tumor and Child-Pugh classification, known as the two key prognostic factors for HCC. However, TrxR still was a prognostic marker even after correcting for those two factors. Unique mechanism should be considered. Firstly, in patients with HCC, it is believed that the main cause of disease recurrence or death is from venous invasion [21], intrahepatic metastasis [38] or metachronous, multi-centric carcinogenesis [39]. Thus, it is likely that the high recurrence rate or death among patients with elevated serum TrxR levels may be due to intra-hepatic metastasis and venous invasion. One explanation for this hypothesis is that patients with high serum TrxR levels already may have cytologic tumor spread that cannot be detected either by routine imaging studies or by pathologic examinations. Another explanation is that TrxR could foster tumor growth [40]. Given the significant correlation between a high serum TrxR level and tumor size or vascular invasion, it is easy to expect that HCC cells already had been released into the portal vein and systemic circulation before treatment. Secondly, TrxRand APE/Ref-1 cooperate in the control of basal p53 activity [41], which is the enzyme present in post mitotic cells and required for dNTP synthesis for DNA repair and mitochondrial DNA synthesis and turnover. Thirdly, most cancer cells have a high level of expression of TrxR, which has been assumed to be a protection against apoptosis and oxidation stress [42]. Because of its role in stimulating cancer cell growth and as an inhibitor of apoptosis [43], TrxR offers a goal for the development of drugs to treat and prevent HCC. Blocking cancer cell DNA replication and repair and induction of oxidative stress by the inhibition of TrxR systems were suggested as cancer chemotherapeutic strategies [44]. Thus, TrxR might play important role in the process of HCC rather than just was a prognosis marker.

Several limitations of this study should be noted. First, in the present study, the classical determination of TrxR by ELISA method is widely used, which is considered ‘gold standard’. However, it is widely known that human serum contains immunoglobulins that inhibit the results of immunoassays by binding to reagent antibodies used in the assay. HAb interference can frequently cause a false positive signal. Our study does not include any additional approach to measure TrxR and validate the findings in at least a subset of patient. Further works in other samples should be considered in the future. In addition, in our study, the levels of TrxR included TrxR1 and TrxR2. We did not get the serum levels of TrxR1 and TrxR2. So we could not determine the association of those factors with HCC outcomes. Second, we only measured TrxR, which is a single antioxidant defense parameter. Effective antioxidant protection is provided by the cooperative and sequential actions of several antioxidant enzymes, and non-enzyme antioxidant molecules. So we could not determine the association of those factors with TrxR levels and HCC. Third, this study was a correlative study, which does not provide data on a potential mechanism by which high serum TrxR would contribute to a worse outcome for HCC patients. The specific mechanisms should be explained by further experiments. Fourth, we measured TrxR in serum, not in histologic specimen. It is still uncertain whether peripheral TrxR levels reflect similar changes in the liver tissue. However, we did not get relevant information in this study. The relationship between peripheral and tissue TrxR levels warrants further investigation. In the future, the more interesting question would be whether TrxR is pathogenically involved in the development of early recurrence and poor prognosis. Lastly limitation was that TrxR measurements were performed after the HCC and may not accurately reflect pre-HCC exposure. Those works should be considered in the future.

## METHODS AND PATIENTS

### Patients

All consecutive patients underwent curative hepatic resection for HCC at department of gastrointestinal surgery of the First Affiliated Hospital of Chongqing Medical University from January 2007 to December 2010 were enrolled into the study. None of the patients had received any previous anti-cancer treatment. Curative resection was defined as complete excision of the tumor with clear microscopic margin and no residual tumor shown by computer tomography scan at 1 month after surgery. In addition, 300 age and sex matched controls (100 patients with liver cirrhosis [LC], 100 patients with chronic liver disease [CLD], and 100 healthy individuals) were also included.

The diagnosis of HCC was made based on guidelines from the Chinese Society of Hepatology, the Chinese Society of Infectious Diseases and the Chinese Medical Association [45–46], and then was also based on tissue pathology. The diagnostic process included routine laboratory tests, serum alpha-fetoprotein (AFP) measurements, and abdominal ultrasound, contrast-enhanced spiral computed tomography, or magnetic resonance imaging. The diagnosis of cirrhosis was based on histology or concordant laboratory and imaging findings. A liver biopsy may be obtained to confirm the diagnosis. The healthy subjects with normal liver biochemistry, no history of liver disease or alcohol abuse, and no viral hepatitis were enrolled from the Health Physical Examination Center of our hospital. This study was approved by the Institutional Review Board of the First Affiliated Hospital of Chongqing Medical University. Written informed consents were obtained from each inductee in accordance with the Ethics Committees Guidelines for our institution.

### Clinical variables

Clinical data collected included age, sex, Child–Pugh score, viral status (hepatitis B virus [HBV] and/or hepatitis C virus [HCV]), and infection time, number of tumors, treatment history, tumor size, venous invasion, tumor node metastasis (TNM) staging, portal vein thrombosis (PVT), etiological risk factors, histological findings and radiological extent of disease. In addition, serum AFP, aspartate aminotransferase (AST), alanine aminotransferase (ALT), total bilirubin (TB), albumin, white blood cell count (WBC), platelet count (PLT), prothrombin time (PT) and high sensitivity C-reactive protein (Hs-CRP) were also collected. The tumor size was the largest diameter measured by imaging. Venous Invasion was referring to the presence of tumor emboli or tumor masses within veins, which was diagnosed by ultrasonography. Patients were classified into the three (A/B/C) Child–Pugh's grades based on their clinical state [47]. Tumor staging (I, II, III, IV) was established using the American Liver Tumor Study Group modified TNM staging classification [48]. Patients with venous invasion should be classified as stage III or more serious.

### Patient follow-up

In our study, the time of follow-up was 36 months. For the first 2 years after the hepatectomy procedure, the HCC patients in our cohort were monitored every 3 months using liver function tests, measurements of the tumor markers AFP and TrxR, and also by ultrasonography and dynamic CT. If recurrence was suspected, both CT and magnetic resonance imaging (MRI) were performed and, if necessary, CT during angiography and bone scintigraphy was undertaken. For the 2–3 years, the time interval was 6-month. Overall survival (OS) was defined as the interval between surgery and death or the last observation taken. For surviving patients, the data were censored at the last follow-up.

### Laboratory testing

The preoperative serum AFP and TrxR levels were simultaneously measured in the patients using standard methods at 1 day before the hepatectomy. Venous blood samples were taken in the morning's fasting state. After at least 30 min, but within 2 h, the tubes were centrifuged at 20°C for 15 min at 1,200 g, and the sera were stored frozen in plastic vials at −80°C until the time of consecutive analyses. The controls samples were collected and stored in the same way as the HCC samples. AFP levels were measured with commercially available immunoassay methods by DPC Immulite 2000 (Diagnostic Products Corporation, CA, USA). A cut-off value of 20 ng/mL was used. AFP level greater than or equal to 20 ng/mL was defined as positive. Serum levels of TrxR was measured in duplicate using a solid-phase sandwich ELISA that uses two highly specific antibodies to human TrxR protein (BioVision Incorporated, Milpitas Boulevard, Milpitas, CA, USA) according to the manufacturer's instruction. The coefficients of variation (CVs) of inter-assay and intra-assay were 5.3–8.6% and 6.6–9.5%, respectively. The lower detection limit was 0.4 U/ml and the line range was 0.4–100 U/ml. Other biomarkers were also tested by standard laboratory method. For all measurements, levels that were not detectable were considered to have a value equal to the lower limit of detection of the assay.

### Statistical analysis

Results are expressed as percentages for categorical variables and as medians (Interquartile ranges, IQRs) for the continuous variables. Univariate data on demographic and clinical features were compared by Mann-Whitney *U*-test or Chi-Square test as appropriate. Correlations among continuous variables were assessed by the Spearman rank-correlation coefficient. In addition, associations between TrxR and tumor size and tumor node metastasis (TNM) staging were also assessed using ordered logistic regression models in multivariate adjustment with possible confounders, such as, sex, age, etiology, family history, HbsAg, viral copies, PVT, venous invasion and serum levels of ALT, AST, TB, PT, AFP and Hs-CRP.

To investigate whether TrxR allows predicting of both recurrence and death 36 months after curative resection, different statistical methods were used. First, the relation of TrxR with the two end points was investigated with the use of Cox regression models. Therefore, common logarithmic transformation (ie, base 10) was performed to obtain normal distribution for skewed variables (ie, TrxR concentrations) as the resulting model yielded smaller Akaike Information Criterion, which was chosen to compare the results. We used crude models and multivariate models adjusted for all significant outcome predictors and report hazard ratios (HR). For multivariate analysis, we included confounders, known risk factors, and other predictors as assessed in univariate analysis. Note that the HR corresponds to a one-unit increase in the explanatory variable. Results were expressed as adjusted HR with the corresponding 95% confidence intervals (CI).

Second, we compared different prognostic risk scores from different predictive models by calculating receiver operating characteristic (ROC) curves analysis. In statistics, a curve is a graphical plot that illustrates the performance of a binary classifiersystem as its discrimination threshold is varied. The curve is created by plotting the true positive rate against the false positive rate at various threshold settings. The ROC curve is thus the sensitivity as a function of fall-out. In this study, ROC was used to test the overall prognostic accuracy of TrxR, AFP and other serum biomarkers and results were reported as area under the curve (AUC). To test whether the TrxR levels improves score performance, we considered the nested models with TrxR, AFP and Hs-CRP as compared with those markers only.

Finally, in order to study the ability of TrxR for OS prediction, we calculated Kaplan–Meier survival curves and stratified patients by TrxR quartiles. All statistical analysis was performed with SPSS for Windows, version 20.0 (SPSS Inc., Chicago, IL, USA) and the ROCR package (version 1.0–2), which is available from CRAN repository (http://cran.r-project.org/). Statistical significance was defined as *p* < 0.05.

## CONCLUSIONS

The search of reliable and efficient biomarkers for a prognostic evaluation of HCC is still an open issue. To the best of our knowledge, this is the first study to report the clinically prognosis value of TrxR for HCC in a cohort of Chinese sample. In this study, we found that elevated serum TrxR levels independently predicted worse survival and recurrence in patients with HCC underwent curative hepatic resection. TrxR was utilized as prognostic indicators of HCC which appeared to be more evident when compare with Hs-CRP and AFP. Although the current study suggests that serum TrxR level may be a useful prognostic biomarker, further studies with larger patient populations are needed to validate its prognostic value and determine the optimum cut-off value. Furthermore, the biologic significance of circulating TrxR in HCC patients remains to be clarified.

## Abbreviations

HCC: Hepatocellular carcinoma
TrxR: Thioredoxin reductase
LC: Liver cirrhosis
CLD: Chronic liver disease
TNM: Tumor node metastasis
PVT: Portal vein thrombosis
AFP: a-fetoprotein
Hs-CRP: High-sensitivity-C-reactive protein
AST: Aspartate aminotransferase
ALT: Alanine aminotransferase
HBV: Hepatitis B virus
HCV: Hepatitis C virus
IQR: Interquartile ranges
ROC: Receiver operating characteristics
OS: Overall survival
HR: Hazard ratios
CI: Confidence intervals
